# Projection of gene-protein networks to the functional space of the proteome and its application to analysis of organism complexity

**DOI:** 10.1186/1471-2164-11-S1-S4

**Published:** 2010-02-10

**Authors:** Alexander A Kanapin, Nicola Mulder, Vladimir A Kuznetsov

**Affiliations:** 1 Ontario Institute for Cancer Research, 101 College St., Toronto, Canada M5G 0A3; 2 Bioinformatics Institute 30, Biopolis street #07-01 Matrix Singapore, 138671; 3 Computational Biology Group, University of Cape Town Health Science Faculty, 7925, South Africa

## Abstract

We consider the problem of biological complexity via a projection of protein-coding genes of complex organisms onto the functional space of the proteome. The latter can be defined as a set of all functions committed by proteins of an organism. Alternative splicing (AS) allows an organism to generate diverse mature RNA transcripts from a single mRNA strand and thus it could be one of the key mechanisms of increasing of functional complexity of the organism's proteome and a driving force of biological evolution. Thus, the projection of transcription units (TU) and alternative splice-variant (SV) forms onto *proteome functional space* could generate new types of relational networks (e.g. SV-protein function networks, SFN) and lead to discoveries of novel evolutionarily conservative functional modules. Such types of networks might provide new reliable characteristics of organism complexity and a better understanding of the evolutionary integration and plasticity of interconnection of genome-transcriptome-proteome functions.

**Results:**

We use the InterPro and UniProt databases to attribute descriptive features (keywords) to protein sequences. UniProt database includes a controlled and curated vocabulary of specific descriptors or keywords. The keywords have been assigned to a protein sequence via conserved domains or via similarity with annotated sequences. Then we consider the unique combinations of keywords as the protein functional labels (FL), which characterize the biological functions of the given protein and construct the contingency tables and graphs providing the projections of transcription units (TU) and alternative splice-variants (SV) onto all FL of the proteome of a given organism. We constructed SFNs for organisms with different evolutionary history and levels of complexity, and performed detailed statistical parameterization of the networks.

**Conclusions:**

The application of the algorithm to organisms with different evolutionary history and level of biological complexity (nematode, fruit fly, vertebrata) reveals that the parameters describing SFN correlate with the complexity of a given organism. Using statistical analysis of the links of the functional networks, we propose new features of evolution of protein function acquisition. We reveal a group of genes and corresponding functions, which could be attributed to an early conservative part of the cellular machinery essential for cell viability and survival. We identify and provide characteristics of functional switches in the polyform group of TUs in different organisms. Based on comparison of mouse and human SFNs, a role of alternative splicing as a necessary source of evolution towards more complex organisms is demonstrated.

The entire set of FL across many organisms could be used as a draft of the catalogue of the functional space of the proteome world.

## Introduction

Information content of genome coding sequences unfolds via functions of proteins. Alternative splicing is one of the ways an organism uses for genome manifestation into its proteome. We consider the problem of projection of genetic information into the functional space of the proteome, where the latter is defined as a set of molecular functions performed by proteins. Not all of the functions of proteins manifest themselves at a level of macroscopic phenotype and therefore the notion of redundancy of proteins could arise. However, this may reflect a failure to provide the correct test for the altered phenotype [1].

An inventory of biological functions of protein is documented in resources such as the FunCat [2] and partly in the Gene Ontology [3] and these use biological knowledge. They include a hierarchical list of all known functions performed by biomolecules in a cell. Here we introduce the automated collection and networking of all possible protein functional annotations. The idea of retrieving a set of cellular functions is not completely new, the functional clusters or modules have previously been revealed in prokaryotic cells [4]. The protein modules detected can be attributed to basic metabolic pathways and well-characterized cellular systems on a global scale.

The protein universe is the set of all proteins of all organisms. Recently all currently known sequences were analyzed in terms of families that have single-domain or multidomain architectures and whether they have a known three-dimensional structure [5]. This analysis has shown that growth of new single-domain families in evolution is very slow. Almost all growth comes from new multidomain architectures that are combinations of domains characterized by approximately 15,000 sequence profiles. The major groups of organisms mostly share single-domain families, whereas multidomain architectures are specific and account for species diversity. Due to these findings, it appears the potential protein universe space of evolutionarily allowed sequences is limited [5-9]. Energy configuration also explains the existence of preferred structures or folds among the proteins. The existing structures are more robust to random mutations and therefore are more evolutionary stable. We also consider a limited space of *protein functions* which may exist in a living cell/organism. The confluence of the two concepts, namely limited space of sequences/structures and limited space of functions may provide a new way of studying molecular evolution.

Alternative splicing is a molecular mechanism that produces multiple protein isoforms from a single gene. AS is considered to be a way of proteome diversification to bridge a complexity gap between the relatively low number of genes in a mammalian genome and the variety of functions in a cell. Analysis of possible changes in protein functions introduced by alternative splicing events is also well developed. An abundance of work, such as [10-16], reviews the problem in detail.

The investigation of functional diversity of splice variants [16] is similar to our approach. The algorithm predicts possible mRNA isoforms from genes. The statistics of different GO categories presume that DNA replication and chromosome cycle genes have more protein isoforms than the average. The study is limited, however, by the data preparation approach, which is computational prediction.

An interesting insight into alternative splicing events on the protein sequence level revealed that the size of splicing events follows the power law distribution [15-19] and the majority of isoforms harbour only one or two alterations [15,16]. Authors also examined the splicing events in the context of protein 3D structures and found that the boundaries of alternative splicing regions generally occur in coil regions of secondary structures and exposed residues and the majority of the sequences involved in splicing are located on the surface of proteins.

All of the investigations of proteome function and complexity analysis done to date [4,7-14] are concentrated around domains as structural evolutionary conserved units (amino acid sequences of proteins). Here we investigate a large set of protein functions in living cells despite the domains, which contribute to them.

Recent reviews of evolution of the protein universe [5-9] suggest that the structure of a protein is an entity conserved during the evolution. A structure stands behind a given function. We extend this notion to a domain of protein functions. A tertiary structure of a protein may be achieved via different primary structures. Therefore, we analyze a conservation and acquisition of new functions in multicellular eukaryotic organisms via projection of information content of a genome into the functional space of the proteome by alternative splicing. It may be easier for evolution to acquire a new function by alternative splicing than by creation of a new conservative domain structure.

The FANTOM project [20] and other transcriptome sequencing data (such as H-Invitational [21,22]) present the opportunity to analyze the exact proteomic impact of a given stretch of DNA sequence, and we have used this data in our study.

## Results

### Data sources and statistics of functional labels.

The following organisms have been used for the analysis: *C. elegans* (CAEEL) *D. melanogaster* (DROME), *A. thaliana* (ARATH), *C. intestinalis* (CIOIN), *T. rubripes* (FUGRU), *M. musculus* (MOUSE), *H. sapiens* (HUMAN). The information about alternative splicing variants and sequences has been provided by various sources, listed in the Table 1. About 70% of all proteins can be attributed to keywords using both InterProScan and BLASTP mapping. At the moment there are a limited number of resources providing information about alternative splicing data for a given organism. We use data provided by the FANTOM consortium for human and mouse transcriptome as a case study to investigate the SFN features thoroughly. FLs for the two species with highest occurrences are listed in the Table 2. A description of FLs for human and mouse as well as the description of the common FL reported for the both species are presented in the Additional files 1,2,3. In this paper, the Isoform Protein Set (IPS) represents the protein sets originating from a single given part of the genome or transcriptional unit.

**Table 1:** Database sources and statistics of Functional labels (FLs), proteins, and fractions of poly- and monoform TUs in different species.

Organism	Database	# FLs	#Proteins	Poly/MonoTUs (%)
ARATH	TAIR/RIKEN	2463	27247	6/8920 (0.07)
CAEEL	WormBase	1150	7854	65/4200 (1.55)
DROME	FlyBase	2335	11129	141/7185 (1.96)
CIOIN	Ensembl	2934	12417	505/8567 (5.89)
FUGRU	Ensembl	3306	24245	843/10760 (7.83)
MOUSE	RIKEN/FANTOM	5172	52957	2353/18574 (12.67)
HUMAN	RIKEN/FANTOM	5183	49829	2315/15944 (14.52)

**Table 2:** Top 10 functional labels for human and mouse data sets

Human			Mouse		
Number of occurence	FL ID	Keywords	Number of occurence	FL ID	Keywords

172	FLh5145	RNA-binding	245	FLm4392	Kinase Nucleotide-binding ATP-binding Serine/threonine-protein kinase Transferase
178	FLh4376	Kinase Nucleotide-binding ATP-binding Serine/threonine-protein kinase Transferase	258	FLm4574	Membrane
207	FLh2670	DNA-binding Nuclear protein Transcription Transcription regulation	267	FLm3271	G-protein coupled receptor Membrane Pheromone response Receptor Transducer Transmembrane
303	FLh4819	Metal-binding Zinc Zinc-finger	321	FLm4818	Metal-binding Zinc Zinc-finger
316	FLh4994	Nuclear protein	342	FLm3276	G-protein coupled receptor Membrane Receptor Transducer Transmembrane
324	FLh5132	Ribonucleoprotein Ribosomal protein	394	FLm4993	Nuclear protein
332	FLh3280	G-protein coupled receptor Membrane Receptor Transducer Transmembrane	501	FLm5125	Ribonucleoprotein Ribosomal protein
489	FLh3273	G-protein coupled receptor Membrane Olfaction Receptor Sensory transduction Transducer Transmembrane	902	FLm2595	DNA-binding regulation Metal-binding Nuclear protein Transcription Transcription Zinc Zinc-finger
882	FLh2637	DNA-binding Metal-binding Nuclear protein Transcription Transcription regulation Zinc Zinc-finger	950	FLm4724	Membrane Transmembrane
1011	FLh4717	Membrane Transmembrane	1091	FLm3270	G-protein coupled receptor Membrane Olfaction Receptor Sensory transduction Transducer Transmembrane

### Classification of transcriptional units (TUs)

The set of TUs can be broken down into two groups according to their ability to produce protein isoforms with identical or different functional assignments. We define them as the monoform and polyform group, respectively. The monoform group includes the majority of TUs. The protein sequences produced by these transcriptional units can differ, but all of them have the same single FL assigned.

The number of TUs that produce polyform sequences varies from 0.06 to 15% in different organisms. We estimated the P-values for frequencies of keywords overrepresented in the human and mouse sets; the results are presented in Table 3. The monoform group has a smaller number of terms in comparison to the polyform group. It also has less functional preferences among keywords that could suggest less dependency on the variation of functions. The difference between the two groups can be noticed in the following trends: the monoform group is more bound to ribosomal g-coupled receptors, while the polyform group includes more kinases, nuclear and nucleic acid binding proteins.

**Table 3:** Keyword over-representation statistics. (1) Monoform TUs and (2) polyform TUs.

1.				
Human		Mouse		Keyword

order by p-value	p-value	order by p-value	p-value	

1	7.81E-20	1	4.24E-51	G-PROTEIN COUPLED RECEPTOR
2	1.38E-18	3	4.18E-44	TRANSDUCER
3	2.21E-16	2	1.58E-50	OLFACTION
4	9.54E-09	6	4.64E-07	RIBOSOMAL PROTEIN
5	8.06E-08	4	1.58E-32	SENSORY TRANSDUCTION
6	5.4E-06	7	0.000989	RIBONUCLEOPROTEIN
7	--	5	2.29E-14	RECEPTOR

**2.**				

Human		Mouse		Keyword

order by p-value	p-value	order by p-value	p-value	

1	2.66E-45	2	3.06E-71	ATP-BINDING
2	2.36E-39	1	2.04E-78	NUCLEOTIDE-BINDING
3	5.43E-22	7	4.29E-20	METAL-BINDING
4	3.88E-19	4	3.15E-27	TRANSFERASE
5	6.36E-17	3	1.34E-31	KINASE
6	1.11E-15	9	4.27E-18	TYROSINE-PROTEIN KINASE
7	8.91E-15	5	2.69E-25	SERINE-THREONINE PROTEIN KINASE
8	1.07E-14	8	1.12E-19	HYDROLASE
9	1.05E-10		--	IRON
10	1.25E-10	6	3.08E-24	NUCLEAR PROTEIN
11	--	10	1.44E-13	TRANSCRIPTION

Only the top 10 keywords are included in the polyform statistics

**Figure 1:**
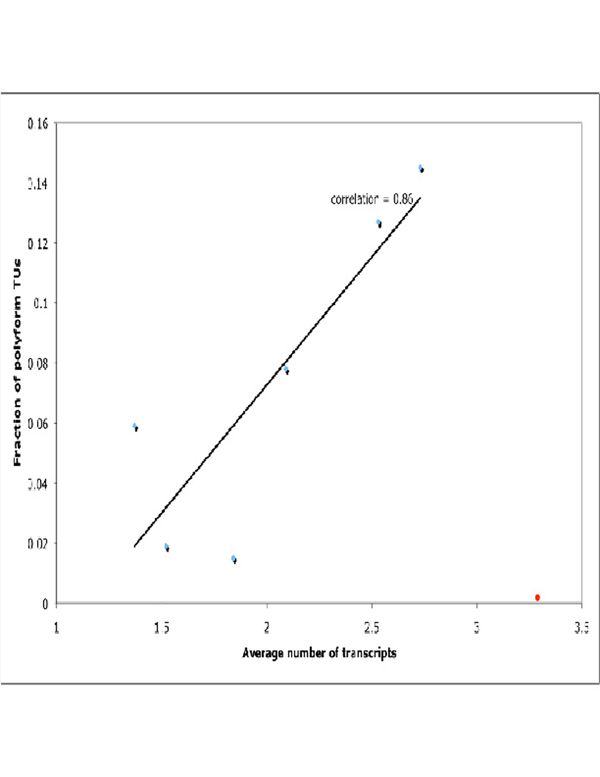
**Correlation between average number of splice variants produced by TU and fraction of polyform TUs.** The linear trend line and correlation coefficient are displayed. The red point represents Arabidopsis data. The Y axis represents the fraction of polyform TUs; the X axis represents an average number of transcripts (splice variants) produced by a TU for a given organism.

Arabidopsis presents an extreme case for the data, in both the statistical and functional results. Figure 1 represents a correlation between the average number of splice variants produces by a TU and a fraction of polyform TUs. There is a good correlation between the data sets, but the Arabidopsis (marked by a red point) is a clear outlier.

### Relationships between the keywords and the number of exons of a given gene

To do this analysis we retrieved from the UniProt database the list of RefSeq Protein IDs corresponding to each keyword. For each Protein ID the corresponding Nucleotide ID was retrieved from UCSC genome database (genome releases hg19 and mm9), along with its exon annotation. Thus, for each RefSeq Protein ID the predicted number of its exons can be obtained. In the proteins annotated by terms G-PROTEIN COUPLED RECEPTOR and RIBOSOMAL PROTEIN and related to monoform TUs, the average numbers of exons per gene are 3.7 and 5.5 for human and 3.8 and 5.7 for mouse, respectively. All these frequency distributions are skewed. The genes with the highest occurrence encode single-exon proteins. The polyform group of genes, represented by the most GO enriched polyform TUs have the keywords NUCLEOTIDE-BINDING, TRANSFERASE and KINASE. In the proteins annotated by these terms, the average numbers of exons per gene are 16.2,13.5,16.5 for human and 14.8, 11.9, 14.7 for mouse, respectively. The frequency distribution of the number of exons in all these cases has gamma-like frequency distribution with maximum nearby average value. These results suggest that differences between the monoform and polyform groups of TUs can be strongly associated with* distinct* structural complexity of the genes and its products.

### Functional switches in the polyform group of TUs

The function of protein isoforms derived from the same TU could differ significantly. We searched through the polyform group for TUs which may be potential candidates for such functional switches. The Jackard distance between sets of keywords assigned to a given TU was calculated. About 0.8% of the TUs analyzed in human and mouse have the distance equal to zero and present candidates for the functional switches. The method can be applied to genome data to reveal potential functional switches among transcriptional units. There are very interesting cases where the protein function changes dramatically, like PA10101 in human switching from protease activity to oxygen transport.

### Shared FLs: functional annotation

**Figure 2:**
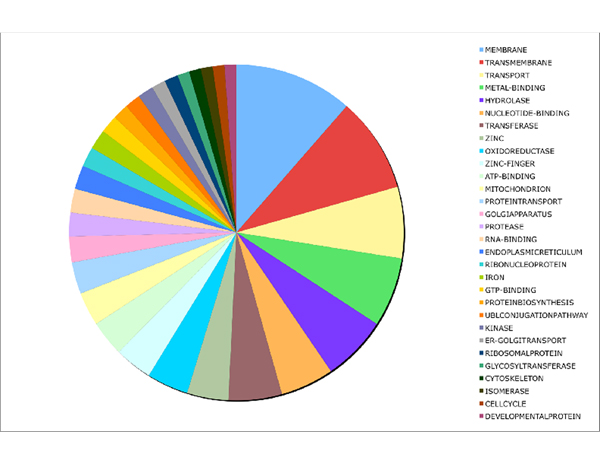
Distribution of the top 30 keywords from the set of TUs shared among all organisms analyzed.

220 FLs are common among all the organisms analyzed. We extracted the top 30 keywords overrepresented in the set of FLs; the diagram is presented in Figure 2. They describe cellular functions corresponding to membrane, transport, receptors, and nucleic acid binding and may be used to deduce a common core of ancient functions acquired by the eukaryotic protogenome.

### Statistics of proteome FLs and transcriptome splice-variants and FL-TU projection events

For detailed analysis of the statistical characteristics of protein function – mRNA relations we focus on data sets for two mammalian organisms: mouse and human. The mouse and human transcriptomes have the best annotations of TUs of the data analysed. We used TUs and splice variant information provided by the FANTOM consortium (see Materials and Methods) and FLs defined by our algorithm. We provided an identification of statistics of three transcript-protein functional relations in the mouse and in the human data sets.

**Figure 3:**
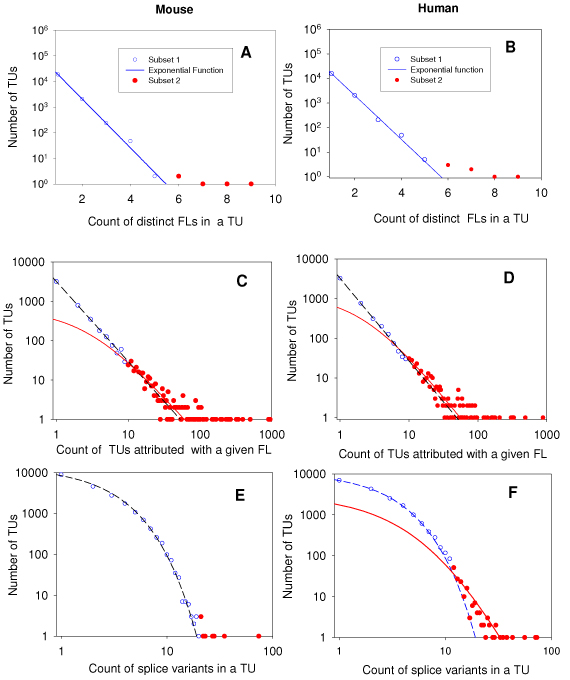
**Best-fit statistics of three transcript-protein relation functions in the mouse (left) and human (right) data sets.** A and B: best-fit frequency distributions of the number of FLs in a given TU; C and D: best-fit frequency distribution of the number of distinct TUs attributed with a given FL in a proteome subset related to selected TUs. E and F: the frequency distributions of the number of splice variant events per TU. The mixture probabilistic model (1) was used for identification of the empirical frequency distributions. Blue symbols: data used for parameterisation of the first model (*P_1_*); blue lines best-fit function *P_1_*. Read symbols: data used for parameterisation of the first model (*P_2_*); blue lines best-fit function *P_2_*. SigmaPlot analytical and graphical tools were used.

First, we found 23640 FLs in the mouse proteome and 20929 FLs in the human proteome. For each of these two sets of FLs, we selected 20928 mouse TUs and 18260 human TUs related to at least one FL in the given TU for mouse and for human, respectively. Note, in our analysis, these TUs can represent 3mRNAs translated to known proteins. After that, we identified a model of frequency distribution of the number of occurrences of FLs in a given TU. Figures 3A and 3B display the empirical frequency distributions of the number of FLs in a given TU in the mouse and in the human data sets, respectively. Both the distribution functions are skewed, for which the most frequent is a single event, however rare events could have occurred on the large dynamical ranges [23,24]. Interestingly, the major fraction (89% (20928/236400)) of mouse TUs translated to proteins exhibits one-to-one relations with corresponding FLs. A very similar fraction of human TUs (87% (18260/20920)) exhibits one-to-one relations with corresponding FLs. The maximum number of distinct FLs in a TU is 9 for both the mouse and the human. The exponential function is fitted well to both the distributions at similar values of exponent constant (Figure 3A &3B; Table 4). However, deviation from simple exponential distribution could be seen on the right tail of the empirical distributions. Note this skewed frequency pattern was observed among all the statistics analysed for other species too (data not presented).

**Table 4:** Descriptive statistics of three studied Transcript-Protein Function relations in mouse and human. m_1_: number of one-to one relationships (singletons), p_1_: % singletons; Skewness: estimated skewness of the empirical frequency distribution.

1.							
**FLs in TU**	**# FLs**	**Total # TUs**	**#FLs /#TUs**	**max # FLs**	**n_1_**	**p_1_,%**	**Skewness**

mouse	23640	20928	1.13	9	18575	88.8	4.05
Hs	20929	18260	1.15	9	15945	87.3	3.99

**2.**							

**TUs attributed with FL **	**# FLs**	**#FL attributes**	**# FLs /# FL attributes**	**Max # FL attributes**	**n_1_**	**p_1_,%**	**Skewness**

mouse	23640	5172	4.58	1091	3198	61.8	25.96
human	20929	5183	4.04	1011	3280	63.3	28.85

**3.**

**# SpVs in TU**	**#SpVs**	**Total #TUs**	**#SpVs/#TUs**	**Max # SpVs**	**n_1_**	**p_1_,%**	**Skewness**

mouse	52957	20928	2.53	74	8920	42.6	4.01
human	49828	18260	2.73	73	6957	38.09	5.55

Secondly, we performed goodness-of-fit analysis of the observed frequency distribution of the number of TUs attributed with a given FL (Figures 3C &3D). For mouse data, 23640 FLs are projected onto 5183 distinct TU attributes and for human data, 20929 FLs are projected onto 5173 distinct TUs (Table 4.2). These results show that the observed sizes of the functional space (total number of TLs ) in mouse and human are very similar, and an average of 3.5 and 4.0 FLs are included in the description of mouse and human TUs, respectively. Taking into account the mostly one-to-one associations between TUs and FLs, these results suggest that the observed sample size of distinct FLs forming functional space is much smaller than the size of the structural space of corresponding TUs.

Figures 3C &3D show the frequency distribution of a given FL in TUs projected on the transcriptome sample of the mouse and human, respectively. These empirical distributions can be well approximated by the standard Pareto discrete function ((2) at *b*=0; [7,8,17,23]) which could be transformed to a linear trend in log-log plot coordinates. However, some additional skewness may be seen at the right tail of both the observed distributions. A goodness-of fit analysis of the right tails of the frequency distributions suggests that an admixture (second model) frequency distribution associated with relatively complex combination FLs (*m*=10,11,…,*J*) might exist. For mouse and human data sets, the second model of the left parts of the frequency distributions is fitted well by the generalized discrete Pareto (GDP) function (2).

Estimates of the distribution slope parameters, (*k+1*), of the individual models of the mixture frequency distribution (Table 4.2) show that statistics of TU complexity (defined by the number of FL in a given TU) in the mouse and in the human proteomes are quite similar. In particular, the slope parameter (*k+1*) of estimated frequency distributions is ~ 2 (known as the Zipf-Lotka law [23]).

The extremely long right tail of the frequency distribution on Figure 3C &3D corresponds to FLs describing the most common functional categories (see Fig.3). The results of Table 4 suggest an admixture of random processes in FL formation. The best-fit parameter *b* in the GDP has the value 2.20 ± 0.004 (p<0.0001) for mouse data and b=2.06 ± 0.015 (p<0.0001) for human data.

Thirdly, for mouse and human, the frequency distribution of the number of splice variants distributed in the set of TUs selected due to association with annotated protein functions is presented in Figures 3E &3F. A skewness of the frequency distribution in the log-log plot is noticeable (Table 4.3). Most events on the left side of the empirical frequency distributions of the number of splice variants can be fitted well by an exponential function with exponent parameter 0.5 ± 0.25 and 0.49 ± 0.006, for mouse and human respectively (Table 5). However, the right part of the frequency distributions of the human data (at m>9 splice variants per TU) has a longer tail and can be fitted well by a GDP function at *k*= 4.16 ± 0.127 and *b*=8.75 ± 0.282 (Table 5). This distribution suggests an appearance of hub nodes in the corresponding functional networks of proteins. Due to the non-zero value parameter *b*, the network is a scale dependent network [8,18]. This indicates an additional mechanistic source of functional complexity of interconnections of splice variants and protein functions in human. 280 TUs inducing 10 or more splice variants could be considered to be a novel confidence TUs sub-set providing specific source of functional diversity of the human proteome versus the mouse proteome.

**Table 5:** A mixture probabilistic model and best-fit parameters of the model for Splice variant-TU relationships based on available mouse and human data

Splice variants-TU's	Mouse	Human
Model 3:P =(1-s)*P1+s*P2		
P1= a* exp(-bm)		
*A*	13898 ± 9509.3	11279 ± 119.9
*B*	0.5 ± 0.25	0.49 ± 0.006
*a*: p-value	0.07	<0.0001
*b*: p-value	0.0836	<0.0001
Std Error of Estimator	3749	48.8
*P2= c/(m+b)^(k+1)*		
*K*	NAN	4.16 ± 0.127
*B*	NAN	8.75 ± 0.282
*k*:p-value	NAN	<0.0001
*b*:p-value	NAN	<0.0001
Std Error of Estimator	NAN	2.7032

### Splice-Function Networks (SFN): statistics and features

We define a Splice-Function Network as a graph built on the basis of a contingency matrix for TUs and FLs. In the analysis we used the polyform group of TUs to build a diagonal matrix of connections between FLs attributed to the TUs. Each element of the matrix is a number of TUs shared between the pair of FLs. Every node in the graph represents a functional label and an edge connecting two nodes corresponds to TU(s), which produce the splice variants attributed to the same FL.

**Figure 4:**
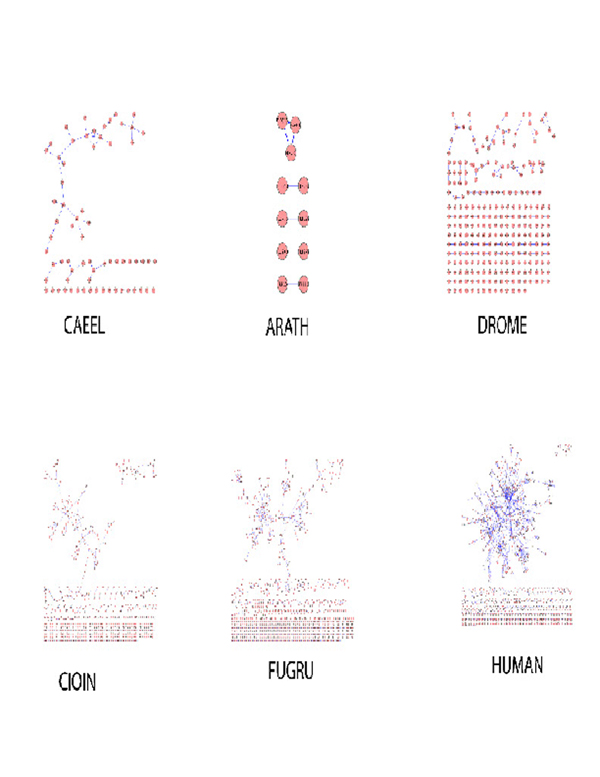
SFN representation for the organisms analyzed

Figure 4 displays the networks for the organisms analyzed. The vertebrata SFNs differ distinctly from others by the following common features: central core of many interconnected nodes; small aggregates of 2-15 nodes and single-node aggregates.

**Figure 5:**
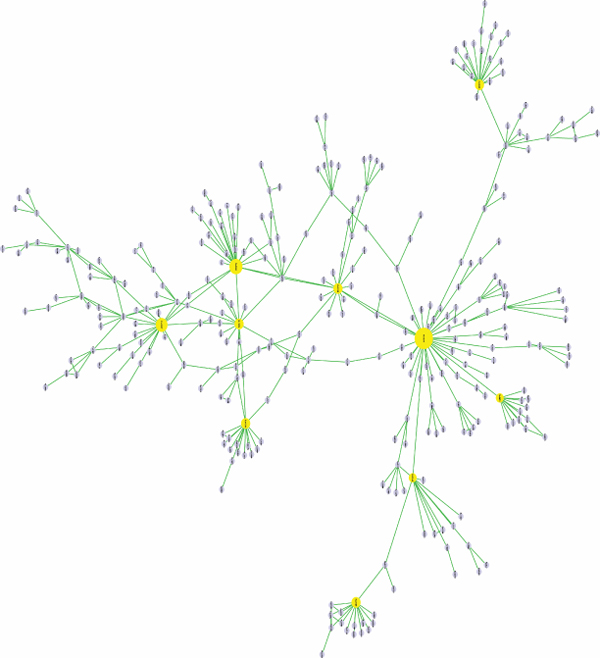
**The biggest cluster in Human-Mouse common FL space.** The size of the nodes is proportional to the number of connecting edges.

Network degree distribution also reflects the differences in the organisms. Vertebrata demonstrate a family of skewed GDP-like distribution functions of SFN links; a shape of these distribution functions corresponds to the appearance of a number of hubs and the connectivity degree interconnecting hubs in the network and correlates with sample size (complexity) of the proteomes. The SFNs for the organisms have common features, which include a central cluster containing about 70% of all nodes, several smaller clusters with 5-20 nodes and an array of single node aggregates (Figure 4). The detailed picture of the central cluster for FLs that are shared between human and mouse is displayed in Figure 5. The size of the nodes is proportional to the number of edges connecting the nodes.

It is interesting that the parameters of the network (the number of nodes, number of edges, etc.) are well correlated with biological complexity of the studied organisms (Table 6).

**Table 6:** SFN statistics and its relation to organism complexity

Organism	#nodes	#edges	Hetero-geneity	Average # neighbors
ARATH	11	7	0.35	1.27
CAEEL	68	53	0.7	1.56
DROME	254	155	0.48	1.22
CIOIN	676	503	0.88	1.49
FUGRU	997	848	1.07	1.7
MOUSE	2562	2594	1.67	2.03
HUMAN	2511	2573	1.89	2.05

We analysed statistics of the distribution of the links between functional units for human and mouse data. The functional labels with the highest number of links are presented in Table 7. They include kinases of different types, transcriptional regulators and DNA and RNA binding proteins. Statistical analysis of the number of links from each node of the graph for both proteomes demonstrates a similar distribution of the empirical data for both sets. The comparison of tables 2 and 7 reveals the functional categories in them are very similar.

**Table 7:** Functional labels with the highest number of links.

FL ID	Number of links	Keywords
Flc3553	11	Membrane
Flc3674	11	Membrane Transmembrane Transport
Flc2789	14	GTP-binding Nucleotide-binding
Flc3759	15	|Metal-binding Zinc Zinc-finger
Flc3914	15	|Nuclear protein
Flc4038	15	|RNA-binding
Flc3398	16	|Kinase Nucleotide-binding ATP-binding Serine/threonine-protein kinase Transferase
Flc2047	17	|DNA-binding Nuclear protein Transcription Transcription regulation
Flc2018	22	|DNA-binding Metal-binding Nuclear protein Transcription Transcription regulation Zinc Zinc-finger
Flc3505	40	|Membrane Transmembrane

Data presented for FLs shared between human and mouse.

## Discussion

We define protein function as an elementary act, which the molecule(s) commits in a living cell. FL is an approximation of the protein function obtained via an automated annotation. The number of FLs varies in the species studied, it has a positive correlation with organism complexity (Figure 4). The number of possible functions picked up by FLs which were found in the mouse and human proteomes are 23640 and 20929, respectively. The numbers of distinct FLs used for the description of these TUs found in the mouse and human proteomes are relatively small (5172 and 5183, respectively; see also Additional files 1-2).

Several types of statistical distributions are observed in our analysis. For instance, the analysis of statistical features of links in the FL - TU interconnection network for complex eukaryotic organisms provides an exponential probability function  describing functional complexity of TUs in terms of FLs. This function fits equally well the mouse and human data set at the same value of exponent parameter. More complex behaviour could be observed in the frequency distribution of the number of distinct biological attributes (keywords) of a given FL in the proteome subset related to studied TUs.

For the mouse and human data, our goodness-of-fit analysis of the left part of the empirical frequency distribution is less complex at m<10: the frequency distribution is fitted well by simple power law (standard Pareto function [23]) with exponent (*k*+1=2). This result assumes the scale-free network statistics function with exponent parameter 2 and shift parameter b=0. Note, similar statistics could emerge in the random graphs when a nodal degree distribution in the graph follows asymptotically (at large enough *m*) GDP function with constant exponent (*k+1*), where *k+1*≈3  and *b*=0 [17]. The distribution function of the events could be described as a stochastic process, which has no or very minor influence on restrictions and/or evolution-driven connections [7-9,18,25-27]. Thus, by a scale-free network model, a new biological function could be acquired or lost by a TU almost arbitrarily with or without selection pressure. This freedom of functional acquisition could have been essential for early biological evolution. On the other hand, one could suggest incompleteness of data and that restrictions and novel interactions could appear in the network when the dataset becomes larger [7,8,19,25].

Another type of relationship was found for statistics of the number of AS isoforms within a TU (Figure 3E &3F). The dynamical range of the number of isoforms per TU is about one order smaller than for the number TUs per FL. The smaller skewness of the distribution of the number isoforms per TU could be associated with restrictions on diversity of isoforms, which could be explained by regulation of alternative splicing itself. The process could not be arbitrary as it is dangerous for a cell. Interestingly, the difference in complexity of proteomes may be reflected by differences between statistics of the number isoforms for human and mouse. This difference is associated with enrichment of more diverse AS for more complex organisms (e.g. human). This suggests that the human reuses domains more frequently in different proteins, and invents more diverse multi-domain proteins than the mouse [7].

The most restricted part of the statistics studied is shown by relationships between between FL and TU (Figure 3C &3D). The long tail of the distribution includes the most common functional categories, presented in Fig.3 and thus may reflect the most conservative part of the proteome functional space, which does not evolve or evolves very slow. The left part of the distribution, in contrast, represents a subset of unique or rare functions and therefore varies between human and mouse data. The more complex organism contains more variable sets of protein functions to reveal the complexity. Table 6 shows that there are 2.5 and 2.7 protein isoforms per TU for the human and mouse proteome, respectively, which are consistent with recent estimates [15,21]. The authors in [15] analyzed a smaller data set from the SWISS-PROT database and revealed that 4,399 proteins can be documented with 8220 protein isoforms averaging about two isoforms per protein. Authors in [21] have identified 18297 unique AS variants associated with a 6877 loci representative data set of the human genome (2.7 variants per loci), made up of 37670 alternative splicing exons (2.1 exons per variant).

Our results confirmed previous observations ([10,14]) that genes can be classified according to their ability to change the function of encoded proteins via alternative splicing. The majority of TUs (above 85%) do not change the function at all. We could conclude that in ~50% of mouse and human proteins, AS modulates proteins and perhaps could essentially modulate protein interconnection networks.  These facts may highlight the functions in the cell, which require steady-state maintenance. For example, household functions can be encoded without the use of alternative splice regulations. In this case, different splice protein isoforms having equal functions could also provide the necessary concentration of the protein product in the cell. As we observed, the monoform group contains nucleic acid-related functions, such as nucleosome, ribosome, transcription regulation, etc., which means the proteins could be produced in large amounts for the important functions of the cell. In contrast, the polyform group contains functions such as enzymatic activities, ion channels, receptors and regulators (Table 3). In this case the idea of building blocks of exons could be proposed. Indeed, our results suggest that differences between the monoform and polyform groups of TUs can be strongly associated with distinct structural complexity of the genes and their products.

We suggest from the comparison of Tables 2 and Table 7 that the most common functions are at the same time the most linked ones in alternative splicing events. Both sets include kinases, transport proteins, signaling pathways, nucleic acid binding and regulation of transcription. A high frequency of AS in kinases genes in human genome was reported in [26]. They belong to the central cluster of the functional network and may represent a snapshot of evolutionary processes in the core part of protogenome, containing important regulatory and transport functions, which, as we discussed above, may be essential for organism complexity. Thus reshuffling of the common blocks, responsible for these functions via alternative splicing events could provide material for evolutionary selection.

The polyform group contains alternatively spliced products with altered functions. Whereas the number of functions of proteins increases, the number of polyform TUs follows the trend. The highly demanded functions then become hubs in the SFN and provide connectivity. The network also becomes more stable and less sensitive to removal of such hubs (data not shown). In some cases the domains are similar, but in different combinations, while in others, the domains differ considerably, resulting in diverse functions. TUs with a high level of functional homogeneity may be reusing certain sets of domain combinations, but with domain substitutions in certain positions to provide a small variation in function, for example, substrate or binding specificity. Higher eukaryotes tend to have multi-domain proteins, which provide flexibility in accessing more functions simply by alterations in domain combinations [7,18,27]. Those TUs with no functional homogeneity are likely to use alternative splicing with mutual exclusivity, potentially using certain exons only in certain isoforms. This would result in isoforms having fewer exons, and thus fewer domains in common. Alternative splicing of this extreme shows the power of the process to increase genetic diversity and complexity without increasing genome sequence complexity.

 The connections between functional labels via hub TUs provide insights into the problem of proto-genes. The central cluster of interconnected FLs on the graphical view of functional space transitions can be considered to be a candidate for the set of functions encoded by the ancient proto-genome. The transition from one function to another can be achieved via common sets of exons. The functions could be encoded by ancient genes, sharing a common pool of exons, which keep the relationships to each other undetectable by other methods of analysis. The transitions between different functions can also represent evolutionary plasticity of the genome where the same function can be achieved in different ways. The absence of correlation between amino acids similarity of protein domains and the number of transcriptional units connecting two functional labels also confirms this idea.

## Materials and methods

### Data Set

The source databases are described in the table 1. We used FANTOM3 RTPS (representative transcript and protein set) for mouse (Fantom3 RTPS 2004-10-27 build) and human (Human RTPS 2004-10-17 build). The datasets are available at ftp://fantom.gsc.riken.jp/RTPS as well as DNA sequences for corresponding transcriptional units. Details of the RTPS production pipeline can be found in [28]. IPS for mouse and human protein sets were used, which contain 73559 and 72047 sequences respectively.

### Keyword and number of exons

To study the relationships between the keywords (G-PROTEIN COUPLED RECEPTOR, RIBOSOMAL PROTEIN, NUCLEOTIDE-BINDING, TRANSFERASE, and KINASE) and the number of exons of a given gene we retrieved from the UniProt database the list of RefSeq Protein IDs corresponding to each keyword. For each Protein ID the corresponding Nucleotide ID was retrieved from UCSC genome database (genome releases hg19 and mm9), along with its exon annotation. Thus, for each RefSeq Protein ID the predicted number of its exons was obtained.

### Functional label mapping

**Figure 6:**
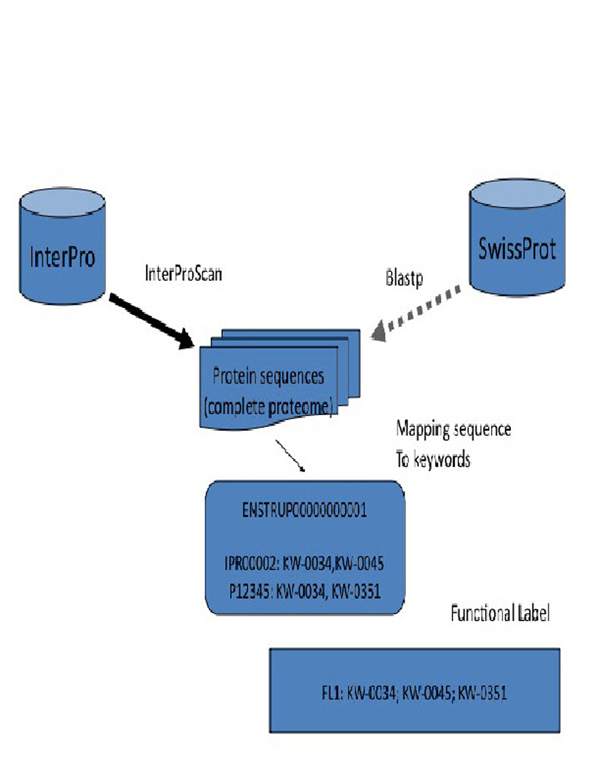
**Data flow in the Functional Label generation algorithm.** The diagram describes a general approach to the FL generation for a given protein sequence (splice variant) via conserved domains and sequence similarity.

A dataflow diagram of the functional label generation is presented in Figure 6. The functional labels were assigned to protein sequences using combinations of UniProt keyword IDs. We used InterProScan version 4.0 and InterPro database [29] version 18.0. InterProScan provides results only for complete matches for InterPro entries; therefore the result set is free from fragments and partial matches. The proteins were scanned against the SwissProt database using BLAST software and the corresponding keywords were retrieved for all exact matches with SwissProt sequences. All the redundant keyword IDs have been removed from the combination leaving only unique keywords for each protein sequence. 

The functional label is *a set* of keyword IDs assigned to a particular protein. We collected and sorted all the combinations and assigned unique numbers for each of the marks separately for human and mouse proteins. The following notation has been used: FL[h,m,c]XX where h,m,c corresponds to FL set – human, mouse or common and XX – unique number.

### Keyword and GO term statistics

We used GeneMerge software [30] to calculate p-values for overrepresented keywords and GO terms for different groups of TUs. The threshold for selection of the overrepresented terms was set to 0.0001. Rank scores for functional or categorical overrepresentation within the study set of genes is obtained using the hypergeometric distribution. The program uses the modified Bonferroni correction for p-values due to multi-dimensional nature of the data.

### Building of contingency matrix and networks graphs

To study the relationships between TUs and protein functional space, we constructed basic contingency tables, which show the responses of FL as a function of TU translation. Non-zero elements of the table indicate co-incidence of events belonging to the both sets. Such a table can provide complete statistical characterization within and between events of both data sets. We created the contingency table for both species and then derived FL–TU–FL links from it. The relations have been used for graphs building with the Cytoscape software.

### A mixture probabilistic model

We assume that the probability distribution function of structural-functional interconnection events (e.g. the number of distinct FLs associated with a given protein translated in TU) could be modeled as a sum of two distributions:

(1) *P(X=m)=* s*P_1_*(*X=m*) + (1-s)* P_2_*(*X=m)*,

(1)

where *P* is the mixture probability distribution function of the interconnection events, *X* is the random number of events; *m*=1,2,3,...,J, J=max{m}. *P_1_* is the probability distribution function of “low-complexity” events (belonging to set 1), 0<s<1 is the fraction of events derived by function *P_1_*. *P_2_* is the probability distribution function of occurrences of “high-complexity” events (belonging to set 2).

We model *P_1_* using the exponential function, standard discrete Pareto function (power law) or the generalized discrete Pareto probability distribution function. We estimated parameters of the functions *P_1_* and* P_2_* and the weight parameter α using algorithm published in [31].

### The Generalized Discrete Pareto Probability Function

The Generalized Discrete Pareto Probability Function [18,19] is used for parameterization of skewed long-tail empirical frequency distributions of our study. It is written by the following:

(2), (2)

where the *f(m)* is the probability that a randomly chosen object occurs *m* times in the entire sample. The function *f* involves two unknown parameters, *k*, and *b*, where *k*>0, and *b*>1;  the normalization factor  is the generalized Riemann zeta-function value:. Note that *J*, the maximum observed abundance, may be a *sample-size* dependent quantity *J=J(M) *[18,19]. The methods of estimation of parameters of the function (2) are presented in [19].

### Statistical methods and Software

We used descriptive statistics and tests provided by SPSS-13 and StatXact-7 software, and goodness of fit analysis and graphical tools from SigmaPlot-8.

## Competing interests

The authors declare that they have no competing interests.

## Authors' contributions

AK developed algorithms, carried out the sequence and network analysis and contributed to the discussion. VK developed statistical analysis algorithms, carried out the statistical calculations and contributed to the discussion. NM participated in the discussion and data analysis and helped to draft the manuscript. AK and VK drafted and revised the manuscript. All authors read and approved the final manuscript.

## Supplementary Material

Additional file 1

Additional file 2

Additional file 3
